# Total Power Radiometer for Medical Sensor Applications Using Matched and Mismatched Noise Sources

**DOI:** 10.3390/s17092105

**Published:** 2017-09-14

**Authors:** Woojin Park, Jinho Jeong

**Affiliations:** Department of Electronic Engineering, Sogang University, 35 Baekbeom-ro, Mapo-gu, Seoul 04107, Korea; woojin8931@naver.com

**Keywords:** detector, microwave radiometer, noise, temperature, remote sensing

## Abstract

This paper presents a simple total power radiometer to noninvasively measure the temperature of the human body. The proposed 3-GHz radiometer consists of an antenna collecting the noise power generated by a target, a low-noise and high-gain receiver amplifying the noise power, and a detector converting the noise power to voltage. A single-pole-triple-throw (SP3T) switch is placed between the antenna and the receiver, while a personal computer is used to control the SP3T switch, collect and process the data such as detector output voltages and physical temperatures of the reference noise sources and the target. The fabricated radiometer shows a good performance agreement with a thermometer in the temperature measurement of water from 25.0 to 43.1 °C. For the accurate prediction of the target temperature, the radiometer is calibrated adaptively to the environment and radiometer variations. For this purpose, two reference noise sources (hot and cold) are proposed using matched and mismatched resistors at room temperature. These resistor-based noise sources offer a reliable performance without complex temperature control systems. Furthermore, they can be easily calibrated in real time by periodically measuring the physical temperatures of the resistors. In addition, the logarithmic detector with wide dynamic range is adopted and logarithmically-fitted based on the measurement results instead of linear approximation, which reduces the error caused by the limited dynamic range of resistor-based noise sources. In order to further increase the accuracy, the performance imbalances between ports in the SP3T switch are also taken into account by employing offsets in the radiometer output voltages.

## 1. Introduction

Microwave radiometers can be used for non-invasive thermal measurement of the human body such as core body temperature monitoring [1] and thermal imaging of breast cancer [2]. However, microwave radiometers experience a variation of performance, such as gain and noise figure, caused by variations in the bias supply and ambient temperature, which leads to the increase of measurement errors [3]. Therefore, it is essential to calibrate the radiometer adaptively to these variations.

Dicke radiometers represent a traditional technique that minimizes the effect of gain variation. They balance the noise power from the antenna and reference noise sources using Dicke switches at the input of the radiometers [3,4,5]. Noise injection to the antenna channel was also introduced for null-balancing of radiometers for the same purpose. They show excellent performance for remote-sensing applications, such as radio-astronomy and earth/ocean/atmosphere sensing. However, they are expensive and bulky systems that require a constant reference noise source (or temperature control system), a variable attenuator or diode (to vary the reference noise power), and a circulator or coupler (to inject the noise power) [5,6,7].

In contrast, medical radiometers require a narrow input dynamic range to measure body temperature at a very short distance, so they can be designed using inexpensive and small-size components. For example, a simple medical radiometer was introduced in [8], where the calibration was performed using a single reference noise source. However, this one-load calibration is inaccurate because it assumes that the noise figure of the radiometer does not change.

In contrast, both the gain and noise figure can be fully calibrated in the two-load radiometers [9], where it is essential to develop stable hot and cold noise sources. Hot noise sources are generally implemented with the avalanche diodes [9,10]. Cooled absorbers are a good cold noise source, but require a bulky cooling system [11]. Active cold noise sources were designed using gallium arsenide (GaAs) pseudo-morphic high electron mobility transistor (pHEMT) technology, which is expensive to fabricate, in [12,13]. Cooled or heated passive resistors or waveguide terminations can also be used as reference noise sources. However, they require additional temperature control systems such as a thermostat or a Peltier controller for reliable performance [6,11].

In this work, a microwave radiometer is proposed for medical applications using the matched and mismatched resistors at room temperature as hot and cold noise sources. These resistor-based noise sources allow sufficient accuracy for medical applications within a relatively narrow input temperature range [9]. The radiometer is calibrated adaptively to the variation of the ambient temperature by measuring the temperature of the resistor noise sources in real time [14]. It operates in the input temperature range from 246 K to 316 K in the experiment.

High-sensitivity logarithmic detectors, approximated as linear for easy calibration, can be used in the radiometers [15]. However, this can increase errors when the input temperature deviates from those of the reference noise sources. In this work, output voltage versus input power of the detector is measured and curve-fitted into a logarithmic function to allow higher accuracy over a wider range of input temperatures. Performance imbalances among ports in the single-pole-triple-throw (SP3T) switch used to select a port (among reference loads and antenna) can also cause an error in the temperature prediction [9]. Such errors are minimized in this work by employing offset voltages of the radiometer output voltages.

A 3-GHz radiometer with two reference loads for medical applications is presented in Section 2, including a two-load calibration procedure. In the same section, hot and cold noise sources are proposed using the matched and mismatched resistors at room temperature. Then, the gain calibration using a logarithmic detector is presented, followed by the port imbalance calibration of the switch and the antenna design. In Section 3, the experimental setup is explained. Finally, the measurement results of the temperature of heated water using the proposed radiometer are presented in Section 4.

## 2. Design of Radiometer

### 2.1. Radiometer with Two Reference Noise Sources

Radiometers are basically low-noise and high-gain receivers that amplify input noise power generated by an object. The output power of the receiver ($P_{OUT}$ (W)) is given as

(1) $$P_{OUT}=G_{REC}kB\left(T_{IN}+T_{REC}\right),$$

where $T_{IN}$ (K) and $T_{REC}$ (K) are the input noise temperature and the equivalent noise temperature of the receiver, respectively [4]. k is Boltzmann’s constant ($1.38\times 10^{-23}$ J/K) and B (Hz) is the bandwidth of the receiver. $G_{REC}$ (W/W) is a receiver gain, which should be accurately calibrated and updated according to the variations in the radiometer and the measurement environment.

**Figure 1:**
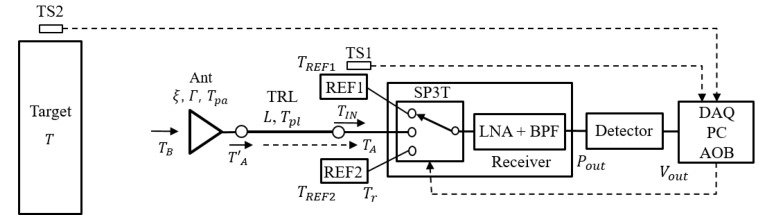
Designed total power radiometer with two reference loads.

Figure 1 shows the designed radiometer in this work with two reference noise sources. It is a total power radiometer consisting of a low-noise amplifier (LNA) and a band-pass filter (BPF). It is a direct-conversion receiver, which is much simpler than super-heterodyne configurations [16]. A detector at the output generates the voltage ($V_{OUT}$ (V)) which is proportional to the input noise power. The output voltage is, then, read through a data acquisition board (DAQ) and processed by a personal computer (PC). Two reference noise sources are used to calibrate the receiver gain $G_{REC}.$ In this work, cold and hot noise sources (REF1 and REF2) are implemented using the mismatched and matched loads at room temperature, respectively. Their operating principle will be explained in detail in Section 2.2. The SP3T switch selects the receiver input among two reference noise sources and the antenna. The analog output board (AOB) generates the switch control signals. The temperature sensors TS1 and TS2 measure the physical temperatures of the two reference noise sources and the target (T), respectively. All the components in the receiver are mounted on a single aluminum jig, so that it is assumed that the physical temperatures of the two reference loads ($T_{r}$), antenna ($T_{pa}$), and transmission line ($T_{pl}$) are all equal.

A square-law detector is commonly used in the radiometers, because it is a linear detector in which the output voltage ($V_{OUT}$) is linearly proportional to input power ($P_{OUT}$ in Figure 1) as

(2) $$V_{OUT}=\gamma_{W}P_{OUT},$$

where $\gamma_{W}$ is the detector sensitivity (V/W). Logarithmic detectors can be assumed as linear for a limited input power range [15,17]. By combining (1) and (2), we obtain

(3) $$V_{OUT}=G_{T}\left(T_{IN}+T_{REC}\right),$$

where $G_{T}$ (V/K) represents total system gain including detector sensitivity, given as $\gamma_{W}G_{REC}kB$.

In order to accurately determine the gain, two-load calibration is employed using cold and hot noise sources (REF1 and REF2). Using the output voltages $V_{OUT1}$ and $V_{OUT2}$ by reference noise sources $T_{REF1}$ and $T_{REF2}$, the total system gain is found as follows.

(4) $$V_{OUTi}=G_{T}\left(T_{REFi}+T_{REC}\right),\;\mathrm{for}\;i=1,2,$$

(5) $$∴G_{T}=\frac{V_{OUT2}-V_{OUT1}}{T_{REF2}-T_{REF1}}.$$

Detailed explanation about two reference noise sources is given later in Section 2.2.

After calibration, the receiver input is switched to the antenna port measuring the target. For detector output voltage $V_{OUT,A}$ for input noise temperature $T_{IN}$,

(6) $$V_{OUT,A}-V_{OUTi}=G_{T}\left(T_{INi}-T_{REFi}\right),$$

(7) $$∴T_{INi}=T_{REFi}+\frac{V_{OUT,A}-V_{OUTi}}{G_{T}},$$

(8) $$∴T_{IN}=\frac{1}{2}\left(T_{IN1}+T_{IN2}\right).$$

That is, average input noise temperature $T_{IN}$ can be obtained from (8) using the determined gain (5) and the measured $V_{OUT,A}$ (6). It should be noted that $T_{IN}$ is a sum of noise temperatures by the target ($T_{A}$), the loss of the transmission line ($T_{TRL}$), and the environment ($T_{ENV}$). $T_{TRL}$ is given as

(9) $$T_{TRL}=\left(1+\frac{{\left|\Gamma \right|}^{2}}{L}\right)\left(1-\frac{1}{L}\right)T_{pl},$$

where L and $T_{pl}$ are insertion loss and physical temperature of the transmission line, respectively [18]. Γ is the reflection coefficient of the antenna.

Two environment-generated noise powers are taken into account: the reflected noise power of the air by the target and the noise power generated by the container of the target (water is used as a target in this work). Therefore, $T_{ENV}$ is given as

(10) $$T_{ENV}=\left(1-\varepsilon \right)\xi \left(1-{\left|\Gamma \right|}^{2}\right)\frac{T_{0}}{L}+\varepsilon^{\prime }\xi \left(1-{\left|\Gamma \right|}^{2}\right)\frac{T_{0}}{L},$$

where ε and $\varepsilon^{\prime }$ are emissivities of the target and the container, respectively. The emissivity can be obtained from Fresnel equations. $T_{0}$ is room temperature and ξ is antenna efficiency [1].

Therefore, input noise temperature by the target only is obtained from

(11) $$T_{A}=T_{IN}-T_{TRL}-T_{ENV}.$$

Then, $T_{A}$ can be used to determine the noise temperature by the target at the antenna output as

(12) $${T^{\prime }}_{A}=\frac{LT_{A}}{\left(1-{\left|\Gamma \right|}^{2}\right)}.$$

Finally, the brightness and physical temperatures of the target, $T_{B}$ and T, respectively, can be determined by the following relations [4].

(13) $$T_{B}=\frac{{T^{\prime }}_{A}-\left(1-\xi \right)T_{pa}}{\xi }$$

(14) $$∴T=\frac{T_{B}}{\varepsilon }.$$

### 2.2. Two Reference Noise Sources

Two noise sources with different noise temperatures are needed for the two-load calibration. In this work, cold and hot noise sources are simply implemented using mismatched and matched resistors for medical applications. The resistor at a physical temperature $T_{r}$ provides an equivalent noise temperature ($T_{REF}$) to a receiver with a reference impedance of $Z_{0}$ as given below [18]

(15) $$T_{REF}=\left(1-{\left|\Gamma_{REF}\right|}^{2}\right)T_{r},$$

where $\Gamma_{REF}=\frac{Z_{0}-R_{REF}}{Z_{0}+R_{REF}}.$ Based on this relation, cold and hot noise sources are fabricated using mismatched ($R_{REF1}=22\;\Omega$) and matched resistors ($R_{REF2}=Z_{0}=50\;\Omega$), respectively. Therefore, $T_{REF1}=0.849\;T_{r}$, and $T_{REF2}=T_{r}$. For example, $T_{REF1}=246$ K and $T_{REF2}=290\;K$ at $T_{r}$ = 290 K. The physical temperature of the resistors $T_{r}$ is measured by the temperature sensor TS1 as shown in Figure 1.

These resistor-based passive noise sources are reliable and stable. In addition, they are compact, easy to fabricate, and cost-effective, compared with other types of noise sources such as diodes, transistors, or temperature-controlled devices. Even when there is a variation in the environment such as room temperature, the noise sources can be calibrated by directly measuring the physical temperature of the resistors and updating the reference noise powers. Therefore, there is no need to accurately control the physical temperature of the noise sources [14].

An SMA load termination was used as a hot noise source. The cold noise source was fabricated using a chip resistor inside an SMA connector which was enclosed by a copper sheet. Its reflection coefficient $\left|\Gamma_{REF1}\right|$ was measured to be 0.4246, on average around 3.0 GHz by a vector network analyzer. It corresponds to $T_{REF1}=0.820\;T_{0}$, where room temperature $T_{0}$ was 296.6 K. This value is slightly different from the theoretical value, which is believed to be caused by the parasitic components of the chip resistor and the copper shielding.

In general, two-load calibrations, hot and cold noise temperatures, are determined to cover the noise temperatures of the target [19]. However, the proposed matched and mismatched noise sources provide a limited range of noise temperatures, for example, from 246 K to 290 K for $R_{REF1}=22\;\Omega$ and $R_{REF2}=50\;\Omega$ at room temperature of 290 K. Furthermore, the target temperature of 307–311 K for a normal human body does not fall into the temperature range of the reference noise sources of this work. In other words, it can be much higher than that of the hot noise source implemented by a matched resistor at room temperature. It can lead to an increase in the error in the temperature estimation of the target, and thus reduce the dynamic range. This issue is alleviated by calibrating the detectors not linearly but logarithmically, as discussed in the following section.

### 2.3. Logaritmic Detector

Basically, square-law detectors with high sensitivity are commonly used in radiometers, since radiometer calibration is based on the linear relation between power and voltage as described in Section 2.1. There are several studies where the logarithmic detector with wide dynamic range was used in the radiometers [15,17]. It can be assumed to be linear in the limited power range for easy calibration. However, in this work, the input noise temperature of a normal human body (307–311 K) can be much higher than that of the hot noise source at room temperature [1]. This fact indicates that input noise temperature does not fall within the range of the two reference noise sources. Furthermore, the detector in this work should cover a wide temperature range from 246 K (of cold noise source) to 311 K. This temperature range can become wider in the case of temperature measurement during hyperthermia therapy for cancer treatment, in which the tumor temperature is increased to 314–318 K [20]. Therefore, temperature estimation errors can increase if the logarithmic detector is assumed linear in this wide temperature range.

In order to achieve higher accuracy, input–output characteristics of the logarithmic detector was measured and modeled using a logarithmic function of

(16) $$V_{OUT}=\gamma \left(10\log P_{OUT}+30\right)+\beta ,$$

where γ (V/dBm) and β (V) are detector sensitivity and offset voltage, respectively.

**Figure 2:**
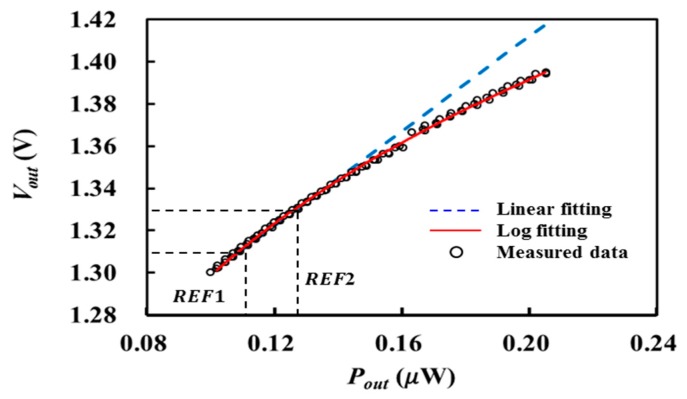
Measured characteristics of the logarithmic detector.

Figure 2 shows the measured output voltage $V_{OUT}$ as a function of input power ($P_{OUT}$ in Figure 1) of the logarithmic detector (LTC5882 by Linear Technology) using a signal generator, a power meter, and a voltage meter. Input power was increased from −40 to −37 dBm at 3.0 GHz. The measurement was repeated four times to increase the modeling accuracy. The least-mean-square method was applied to fit these measurement data to (16), which resulted in γ=0.0308 and β=2.5301. In Figure 2, dots represent the measurement results and the solid curve represents the fitted model. Also included is the linearly-fitted model with a slotted line, which was obtained from the two measured $V_{OUT}$ values at hot and cold reference noise sources. It can be expected for the linearly-fitted model that the errors will keep increasing as the target temperature increases higher than that of the hot source (REF2).

After the logarithmic detector is modeled, the receiver gain $G_{REC}$ is calibrated instead of $G_{T}$ using two reference noise sources. That is, the output powers ($P_{OUT1}$ and $P_{OUT2}$) of the receiver are determined by the inverse function of (16) for the measured $V_{OUT1}$ and $V_{OUT2}$ at cold and hot noise sources, respectively. The receiver gain $G_{REC}$ is obtained as

(17) $$G_{REC}=\frac{P_{OUT2}-P_{OUT1}}{kB\left(T_{REF2}-T_{REF1}\right)}.$$

Then, the receiver is connected to the antenna port and $P_{OUT,A}$ is calculated from the measured $V_{OUT,A}$. The input noise temperature $T_{IN}$ is determined by averaging two $T_{INi}$ given as

(18) $$T_{INi}=T_{REFi}+\frac{P_{OUT,A}-P_{OUTi}}{G_{REC}kB},\;\mathrm{for}\;i=1,2.$$

The target temperature T can be determined from $T_{IN}$ by following the same procedure in (9)–(14).

### 2.4. SP3T Switch Calibration

There is an SP3T switch in the input side of the receiver which selects the port among two reference loads and antenna as shown in Figure 1. It is usually assumed that the SP3T switch exhibits the same characteristics in three throw ports in terms of insertion and return losses. However, real SP3T switches can have performance imbalances among three ports, which leads to inaccuracy in the calibration process. In [9], the insertion loss imbalance of the switch was taken into account in the calibration of the radiometer. In this work, the performance imbalances among the ports are compensated for by adding different offset voltages for each port to generate the same voltage when each port is terminated with the same 50 Ω load.

### 2.5. Receiver Design

**Figure 3:**
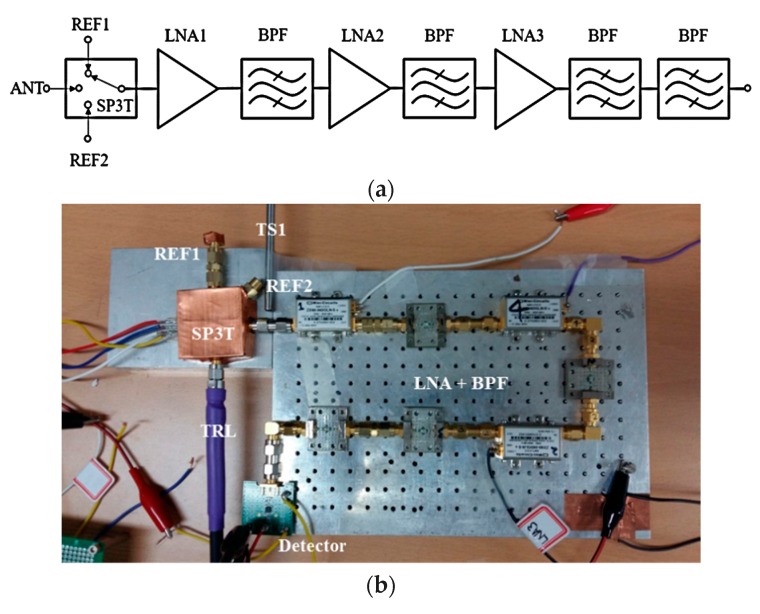
Designed direct-conversion receiver. (**a**) Block diagram. (**b**) Photograph.

The operating frequency of medical radiometers should be carefully determined, with consideration of spatial resolution and penetration depth of electromagnetic waves into human tissues. A low frequency band around a few GHz with deep penetration depth is desirable for the temperature measurement of the core body with thick lossy tissue layers [19]. Figure 3 shows the designed direct-conversion receiver operating at 3 GHz.

This frequency band was used for the radiometric temperature measurement of human breast or brain [8,21]. Three LNAs (ZX60-362GLN+ by Mini-circuits) with a gain of 20 dB and noise figure of 0.9 dB are cascaded to have sufficient gain, and four BPFs with insertion loss of 2.4 dB (BFCN-3010+ by Mini-circuits) are properly inserted between LNAs to limit the bandwidth of the receiver. The SP3T switch (SKY13345-368LF by Skyworks) exhibits insertion loss of ~0.8 dB around 3 GHz.

**Figure 4:**
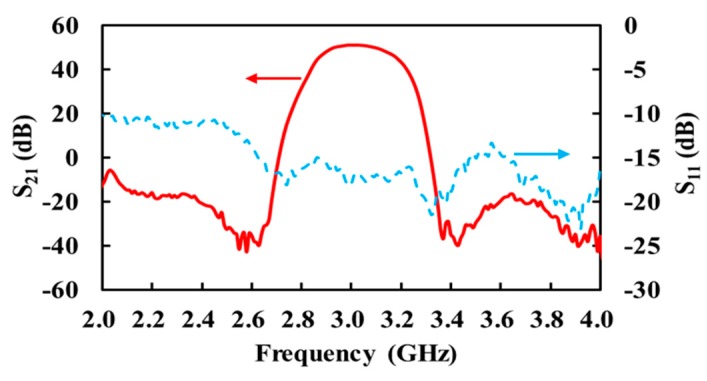
Measured S-parameters of the receiver.

Figure 4 shows the measured $S_{11}$ and $S_{21}$ of the fabricated receiver. It exhibits a 3-dB bandwidth B of 230 MHz from 2.88 to 3.11 GHz, where the gain is 49.8 dB and input return loss is better than 15.3 dB.

### 2.6. Antenna Design

Several antennae were proposed for medical radiometers such as shielded elliptic antenna [17], folded dipole, and patch antenna [10,19]. In [1], noncontact cavity-backed slot antenna was proposed to minimize the near field diffusion which can limit the measurement depth. A rectangular waveguide antenna was used at very high frequency, like a Ka-band [22].

**Figure 5:**
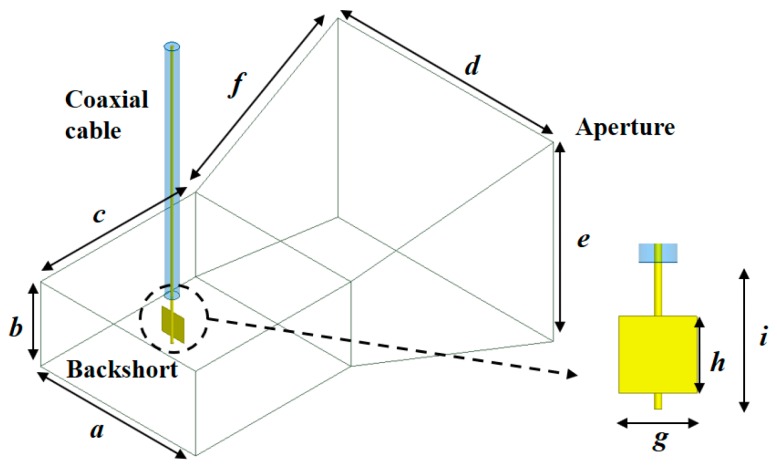
Designed horn antenna with coaxial-feeding.

In this work, a waveguide horn antenna with high directivity was selected for non-contact radiometers, which can reduce the noise contribution from the environment. However, the commercially available horn antenna operating at 3 GHz has a relatively large aperture size, which is undesirable for the real applications. Therefore, the horn antenna was designed by making a trade-off between directivity and aperture size. Figure 5 shows the designed horn antenna which is based on the WR-284 waveguide with coaxial feeding. The flared section (*f*, *e*, and *d* in Figure 5) was optimized considering the directivity and size. The rectangular metal (copper) plate was attached to the center conductor of the coaxial line, which functions as a coaxial-to-waveguide transition.

Table 1 lists the optimized dimensions obtained from the simulation using Ansys HFSS. The designed antenna exhibits a simulated gain of 9.63 dBi, efficiency ξ of 95.4%, and 3-dB beam width of 60° at 3 GHz.

**Figure 6:**
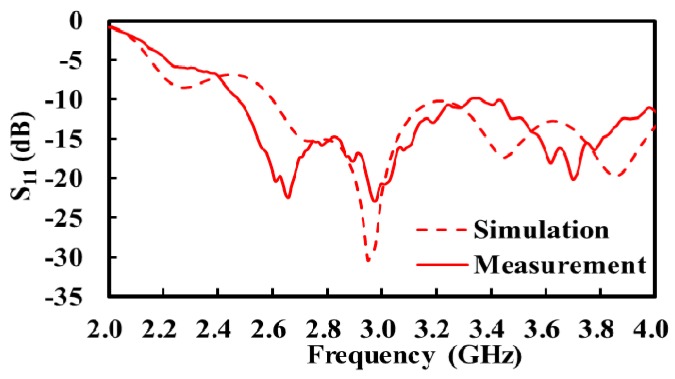
Simulated and measured S_11_ of the fabricated horn antenna.

Figure 6 shows the simulated and measured $S_{11}$ of the fabricated antenna. The measured return loss is better than 15.5 dB across the 3-dB bandwidth of the receiver. The insertion loss L of the antenna cable (transmission line) was measured to be 0.74 dB.

## 3. Temperature Measurement Setup

**Figure 7:**
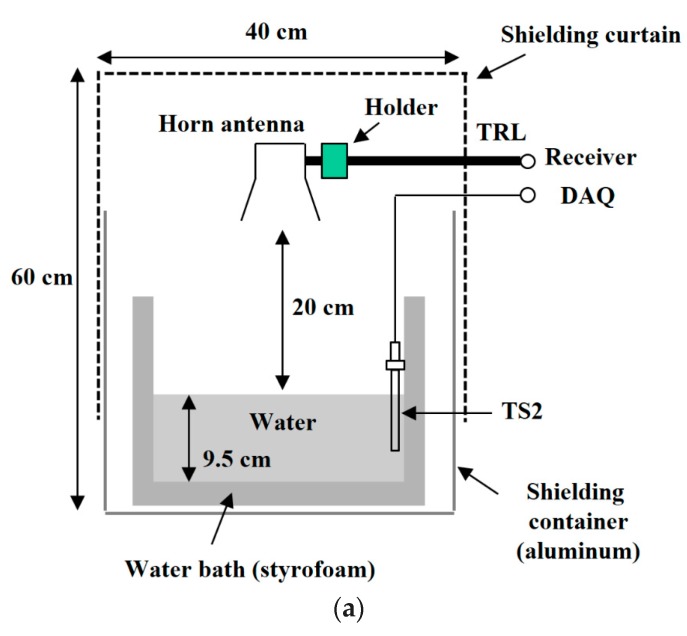

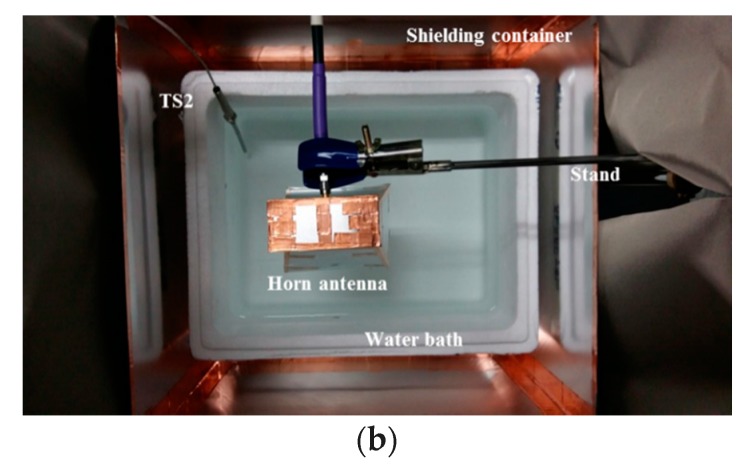
Experimental setup for water temperature measurement. (**a**) Diagram. (**b**) Photograph.

Several measurement setups have been introduced to validate the performance of medical radiometers, such as homogeneous water or NaCl solution benches [6,19], a water bench with a hot object embedded [23], and a multi-layered human tissue phantom [1,8]. In this work, the performance of the designed radiometer was verified by measuring the temperature of the heated water in the bath. The emissivity of water (ε) is calculated to be 0.98 using the Fresnel reflection coefficient at the water/air interface. Figure 7 shows the experimental setup, where the water bath (styrofoam with dielectric constant of 1.03) is covered with a shielding curtain (with 60 dB attenuation) on the top and an aluminum container on the bottom [24]. The horn antenna is placed 20 cm above the surface of the water in order to alleviate the near-field diffusion problem in the contact radiometer [1]. The thermocouple temperature sensors (TS1 and TS2) were calibrated using a high-precision thermometer (BAT-10 by Physitemp).

During measurement, the radiometer was periodically switched among two reference noise sources and antenna by the SP3T switch. Each detector voltage was sampled by the DAQ board and stored in the PC. The sampling rate was 1 kHz. The detector voltages were averaged every 250 samples and used to determine the receiver gain. The increase of the sample size used in averaging can lead to higher accuracy at the cost of time. The moving average technique was applied to the data. More advanced digital signal processing techniques can be applied to further reduce the errors.

## 4. Temperature Measurement Result

**Figure 8:**
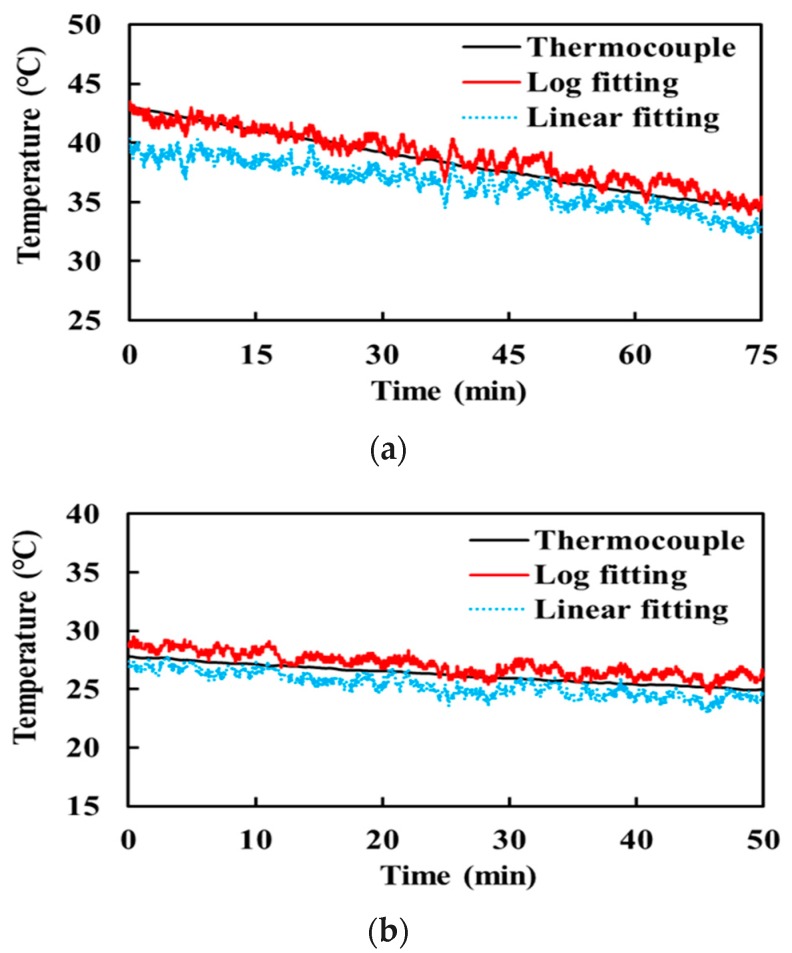
Measured temperature of water by the temperature sensor (thermocouple) and radiometer (**a**) from 43.1 to 34.5 °C and (**b**) from 27.8 to 25.0 °C.

Figure 8 shows the measurement results of water temperature. The heated water was naturally cooled from 43 to 25 °C for 240 min. First, the measurement was taken for 75 min, during which the water temperature decreased from 43.1 to 34.5 °C which covers the temperature range of hyperthermia therapy and of the human body. Then, the measured data was analyzed for 115 min to check if the radiometer worked well. Finally, the measurement was resumed to check the performance of the radiometer in the lower temperature range. The water temperature changed from 27.8 to 25.0 °C for 50 min in this second measurement. The water around room temperature (20 °C) cooled very slowly, so that the measurement was stopped at 25.0 °C.

The figure includes the measured temperature by the temperature sensor (TS2) for comparison. There are two radiometer-measured temperatures depending on the detector modeling: one with linear fitting (dotted curve) and the other with logarithmic fitting (solid curve). This figure demonstrates that the linear approximation of the logarithmic detector can provide a relatively accurate temperature only around the temperature of the hot noise source (Figure 8b). However, the error increases as the target temperature deviates away from the reference temperature, as shown in Figure 8a. On the contrary, the logarithmically fitted detector maintains a high accuracy even in the high temperature range. The linearly fitted radiometer exhibits a measurement error of 1.93 and 0.90 K between 43 and 34.5 °C and between 27.8 and 25 °C, respectively. This error was reduced to 0.62 and 0.85 K, respectively, with the logarithmically fitted radiometer.

**Figure 9:**
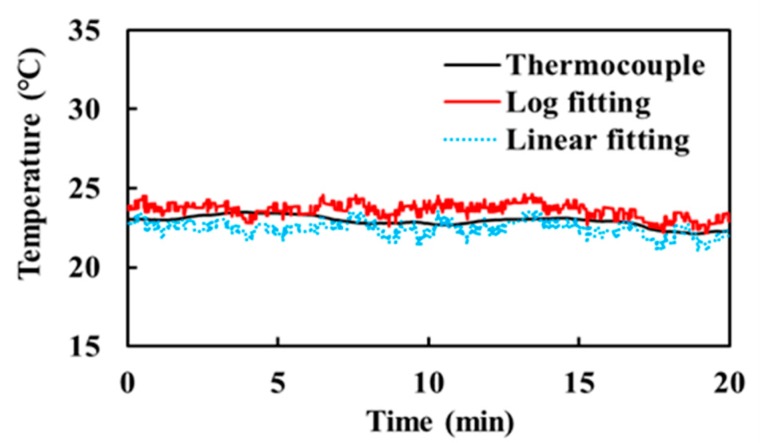
Measurement of water at ambient temperature.

To evaluate the performance of the radiometer for an object around room temperature, water at an ambient temperature of ~23 °C was measured for 20 min. Figure 9 shows the measurement results performed on a different day from the experiment of Figure 8. The measurement errors of the linear and logarithmic fittings are 0.54 and 0.73 K, respectively, which are smaller than those in the heated water experiment. This result indicates that the linearly fitted radiometer becomes more accurate when an object temperature is around the reference noise temperatures. In contrast, the logarithmically fitted radiometer allows a wider range of object temperature.

## 5. Conclusions

This paper proposes a microwave radiometer with simple and reliable noise sources for medical applications. The proposed matched and mismatched resistor noise sources can be easily calibrated in real time by directly measuring their physical temperatures. Therefore, the radiometer does not require a bulky and high-cost temperature control system. In addition, a high-sensitivity logarithmic detector was calibrated without linear assumption, which provided more accurate temperature estimation, even though the target temperature ranges away from those of the reference noise sources. Performance imbalances among ports in the SP3T switch were also calibrated by employing offset voltages in the detector output voltage. The designed radiometer accurately predicted the temperature of water in the range of 25 to 43 °C. This result indicates that the proposed radiometer can be applied for temperature measurement of the human body.

In future work for medical applications, a human tissue phantom will be developed and research will be performed on determining the tissue temperature using the radiometric measurement. The horn antenna designed in this work is still bulky for real applications. For more practical applications, research is being carried out on replacing the horn antenna with a compact planar-type antenna such as spiral antenna.

## Figures and Tables

**Table 1:** Dimension of designed horn antenna.

	*a*	*b*	*c*	*d*	*e*	*f*	*g*	*h*	*i*
Length (mm)	72	34	72	100	80	102.6	10	10	19
